# Role of Optineurin in the Mitochondrial Dysfunction: Potential Implications in Neurodegenerative Diseases and Cancer

**DOI:** 10.3389/fimmu.2018.01243

**Published:** 2018-06-19

**Authors:** Robert Weil, Emmanuel Laplantine, Shannel Curic, Pierre Génin

**Affiliations:** Laboratory of Signaling and Pathogenesis, Institut Pasteur, CNRS UMR3691, Paris, France

**Keywords:** autophagy, mitophagy, autophagy receptor, pathologies, neurodegenerative diseases, cancer

## Abstract

Optineurin (Optn) is a 577 aa protein encoded by the *Optn* gene. Mutations of *Optn* are associated with normal tension glaucoma and amyotrophic lateral sclerosis, and its gene has also been linked to the development of Paget’s disease of bone and Crohn’s disease. Optn is involved in diverse cellular functions, including NF-κB regulation, membrane trafficking, exocytosis, vesicle transport, reorganization of actin and microtubules, cell cycle control, and autophagy. Besides its role in xenophagy and autophagy of aggregates, Optn has been identified as a primary autophagy receptor, among the five adaptors that translocate to mitochondria during mitophagy. Mitophagy is a selective macroautophagy process during which irreparable mitochondria are degraded, preventing accumulation of defective mitochondria and limiting the release of reactive oxygen species and proapoptotic factors. Mitochondrial quality control *via* mitophagy is central to the health of cells. One of the important surveillance pathways of mitochondrial health is the recently defined signal transduction pathway involving the mitochondrial PTEN-induced putative kinase 1 (PINK1) protein and the cytosolic RING-between-RING ubiquitin ligase Parkin. Both of these proteins, when mutated, have been identified in certain forms of Parkinson’s disease. By targeting ubiquitinated mitochondria to autophagosomes through its association with autophagy related proteins, Optn is responsible for a critical step in mitophagy. This review reports recent discoveries on the role of Optn in mitophagy and provides insight into its link with neurodegenerative diseases. We will also discuss the involvement of Optn in other pathologies in which mitophagy dysfunctions are involved including cancer.

## Introduction

Mitochondria play a central role in almost all eukaryotic cells in the production of the energy source, ATP, through oxidative phosphorylation (OxPhos). Besides their metabolic function, mitochondria maintain overall cellular homeostasis by the generation of reactive oxygen and nitrogen species (ROS and RNS) and occupy a central position in the induction of programmed cell death (1, 2). Mitochondria also play a pivotal role in viral sensing, by localizing the key antiviral regulator MAVS. Given the importance of these functions, this organelle must constantly assess its integrity. If damaged, mitochondria can be toxic to the cell and must be rapidly eliminated by a selective autophagy mechanism termed mitophagy. This process is not only needed to eliminate old/damaged mitochondria, but is also used in many other physiological processes, and as anticipated, its deregulation can lead to pathological situations. In this review, we will first describe the different normal functions of mitophagy. We will focus on the molecular mechanism utilized to remove damaged mitochondria, a process dependent on the autophagy receptor Optineurin (Optn) and the E3 ubiquitin ligase Parkin. We will then review the links that have been uncovered between a deregulation of mitophagy and several human diseases. In this part, we will look into the involvement of Optn in these diseases as well as the possible role of mitophagy in Optn-linked pathologies.

### Mitochondria Dynamics

To maintain integrity of their functions, mitochondria engage several dynamic activities, such as biogenesis (generation of new mitochondria), fusion, fission, transport, and mitophagy (destruction by autophagy). Importantly, the energetic state of cells is often associated with specific mitochondrial morphologies. Thus, elongated mitochondrial networks are more efficient at energy generation, while depolarization or hypoxia inhibits fusion, and triggers fission followed by mitophagy (3). As these dynamic activities are intimately associated and share several proteins, each of these processes will be briefly described before detailing the molecular aspects of mitophagy, the process in which Optn is involved.

Mitochondrial fusion is a cell type-dependent event that often occurs in cultured cell types and less frequently in tissues (4, 5). In mammals, mitochondrial fusion is mediated by mitofusin (Mfn)1 and 2, and optic atrophy 1 (Opa1) belonging to the dynamin GTPases superfamily (6, 7). Mitochondrial fusion requires outer-membrane fusion mediated by the integral outer-membrane proteins Mfn1 and Mfn2, followed by inner-membrane fusion involving multiple isoforms of OPA1. Fusion events not only serve to regulate mitochondrial functions but can also prevent harmful defects. Thus, the inability of defective mitochondria to fuse with unaffected organelles constitutes a way to segregate mutant organelles for destruction by the mitophagy process (8). Myosin VI was very recently shown to play an important role in this process by creating cages allowing encapsulation and isolation of damaged mitochondria from the network (9). In addition to regulating the mitochondrial networks and the metabolic processes, fission has been involved in mitochondrial transport, mitophagy, and apoptosis. Division of mitochondria requires the interaction of the large GTPase dynamin-related protein 1 (Drp1) with the receptor proteins (Mff, Fis1, MiD49, and MiD50) that ensure its recruitment to the mitochondrial outer membrane. Subsequently, Drp1 accumulates around the mitochondria tubules and constricts it to mediate scission in a GTP-dependent manner. In mammals, mitochondrial transport requires the activity of motor proteins associated with the microtubule network; however, transport along other cytoskeletal elements can also occur (10). Miro1 and Miro2, two mitochondrial transmembrane GTPases, connecting the motor proteins to the mitochondrial surface, interact with kinesin motors *via* the Milton proteins, Trak1 and Trak2 (11, 12). These Miro–Milton–kinesin complexes insure the anterograde transport of mitochondria along microtubules. In neurons, mitochondria trafficking events are critical for neuronal functions. Whatever the reason for mitochondrial elimination (defective, aged, or excess), clearance occurs essentially by mitophagy. This degradation can be either constitutive or induced, as mitochondrial removal is triggered by metabolic stress as part of the autophagy program induced in cells. Thus, mitophagy can be activated under cellular stresses such as activation of AMP-activated protein kinase (AMPK), leading to phosphorylation of uncoordinated (Unc)-51-like kinase 1 (ULK1) and ULK2, considered as autophagy (and, therefore, mitophagy) initiators (13). AMPK also acts by inhibiting the growth-promoting mTORC pathway that negatively regulates the ULK1/2 function, therefore, providing a link coupling mitophagy to the nutrient status of the cell (14).

### Physiological Functions of Mitophagy

**Figure 1:**
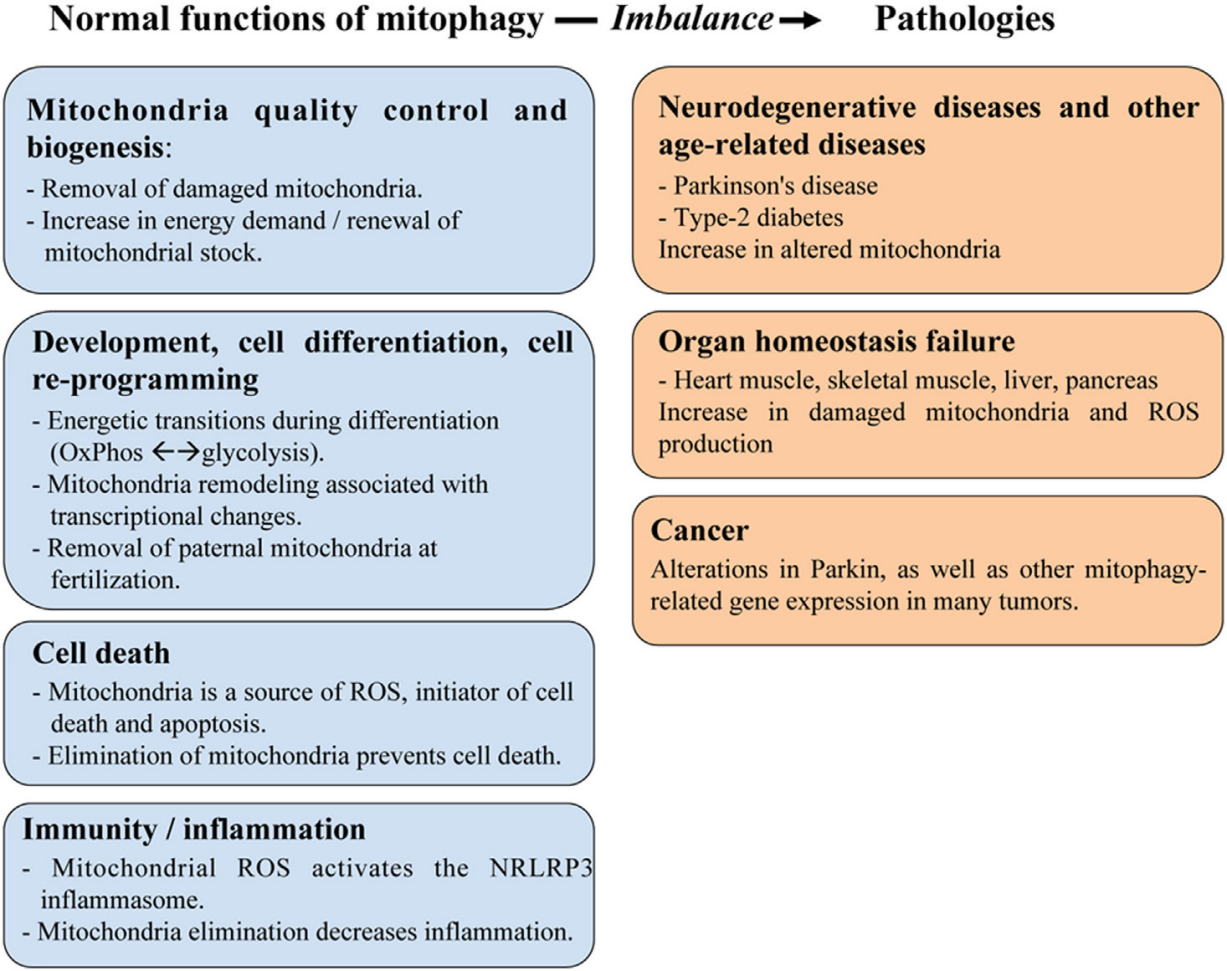
The role of mitophagy in physiology and human pathologies. Mitophagy plays an important role in maintaining mitochondria homeostasis including quality control of mitochondria and cell metabolism, as well as in regulating various aspects of cellular function, such as development/differentiation, cell death, and immunity/inflammation. These roles are critical to prevent developing human diseases linked to organ failure (heart muscle, skeletal muscle, liver, and pancreas) or to age-related dysfunctions (cancer, neurodegenerative diseases).

The primary role of mitophagy is to provide a quality control mechanism allowing the recognition of damaged mitochondria and their selective removal. Consequently, mitophagy is involved in many physiological functions, summarized in Figure 1. Using *C. elegans* as a model, Plaikaras et al. (15) first demonstrated that mitophagy and mitochondrial biosynthesis are inter-connected to maintain mitochondrial homeostasis. This study revealed a metabolic-dependent mechanism that coordinates the biogenesis and turnover of mitochondria. During aging, uncoupling of these two mitochondrial-related activities was proposed to increase the number of damaged mitochondria and to participate in the deterioration of cellular functions. In fact, many studies have reported that mitophagy may act as a crucial precursor of mitochondrial remodeling, such as during myogenesis (16).

Mitophagy operates in numerous developmental and differentiation events, a remarkable example being erythropoiesis (17). During this process, after a rapid proliferation step, immature erythroblasts generated in the bone marrow are first enucleated to form erythrocytes, after which, all remaining intracellular organelles and ribosomes are then eliminated. At this step, the specific destruction of mitochondria occurs *via* mitophagy, which is dependent on the mitochondrial outer membrane protein Nix by connecting the mitochondria to the autophagic machinery. In the Nix-knockout mice, the engulfment of mitochondria into autophagosomes is defective and mitochondria are not eliminated, leading to anemia attributed to the production of reactive oxygen species by persistent mitochondria (18). Mitophagy is also required, during the early embryonic development of *C. elegans*, to selectively eliminate paternal mitochondria from fertilized oocytes (19, 20). Clearance of mitochondria by mitophagy is also required for the differentiation of myoblasts into myotubes, a transition in which energy demand goes from glycolysis to OxPhos (16). An opposite metabolic switch from OxPhos to highly glycolytic is orchestrated during the differentiation of retinal ganglion cells (RGCs). Significant changes in energy metabolism associated with mitochondrial remodeling also take place during cell re-programming. Induced pluripotent stem (iPS) cells have fewer and less mature mitochondria than somatic cells and mostly depend on glycolysis for energy source. Interestingly, recent studies have reported that mitochondrial restructuration and energy metabolism transitions are required for the induction of orchestrated de-differentiation and iPS cells (21).

Furthermore, mitophagy has been associated with cell death and tissue injury. Mitochondria have long been known to play a key role in the orchestration of apoptosis induced by many stress signals, including growth factors deprivation, hypoxia, oxidative stress, and DNA damage. Intracellular pro-apoptotic signals trigger permeabilization of the mitochondrial membrane, leading to the release of apoptosis-inducing factors (such as cytochrome-c and SMAC/Diablo) from the inter-membrane space of the mitochondrial into the cytosol (22). Several studies strongly suggest that upon high-stress conditions, when important mitochondrial damage occurs, activation of apoptotic proteases shutdown autophagy/mitophagy and activate apoptosis to ensure cell death. In addition to apoptosis, necrosis-induced stimuli and tissue injury also promote the onset of the mitochondrial permeability transition, which is accompanied by a loss in ATP synthesis. In the ischemia/reperfusion (I/R) model of heart injury, mitophagy exerts a protective role against the death of cardiomyocytes. A cell-protecting role of mitophagy against tissue injury was also demonstrated during paracetamol- and alcohol-induced liver damage, as well as in steatosis conditions (23). On the other hand, a disrupted or over-activated autophagic flux is implicated in many examples of pathological cell death (24). Identification of the molecular mechanisms regulating the balance between mitophagy and cell death could provide insights into the processes responsible for the maintenance of tissue function and for the appearance of diseases as a result of their dysfunctions.

Autophagy is involved in many signaling networks controlling innate and acquired immunity and deregulation of autophagy networks leads to immune diseases. In addition, mitophagy plays an important role in limiting pro-inflammatory signals induced by either pathogen-associated molecular patterns such as bacterial lipopolysaccharide (LPS), or danger-associated molecular patterns such as extracellular ATP. ROS signaling is an important mediator of the NLRP3 (NLR family, pyrin domain-containing 3) inflammasome activation, and the source of ROS was initially thought to originate from the cytosolic DADPH oxidase. More recent work provided evidence that damaged mitochondria, a major source of ROS, and mitochondrial DNA (mtDNA) are both involved in NLRP3 inflammasome activation (25). At the opposite, inflammasome activation can lead to accumulation of damaged mitochondria, due to caspase-1-mediated degradation of Parkin, and to subsequent inhibition of mitophagy, highlighting the interconnections between the inflammasome and autophagy/mitophagy (26). A recent study further showed that LPS-stimulated macrophages induce the NF-κB-dependent increase in expression of the autophagic receptor p62 that mediates mitochondria clearance and thereby limits inflammation (27). Overall these data suggest that the control of mitochondria integrity is a key factor in the balance needed to clear pathogens, while maintaining tissues and preventing overt inflammation.

Mitochondria also occupy a central place in the innate immune defense against viruses. Infection by several viruses, such as influenza, hepatitis B, hepatitis C, and measles viruses has been reported to stimulate mitophagy. For example, the measles virus of the Edmonston vaccine strain triggers p62-mediated mitophagy to decrease MAVS (mitochondrial antiviral signaling, required for the activation of NF-κB/IRF transcription factors and induction of type I interferon gene expression) triggered antiviral signaling, which weakens the innate immune response (28). Additionally, the matrix protein of human parainfluenza virus type 3 targets the autophagic marker microtubule-associated protein 1A/1B-light chain 3 (LC3) with similar effects (29). Interestingly, CMV virus has also been shown to induce mitochondrial fragmentation, through the action of a CMV-expressed anti-apoptotic protein, leading to the inhibition of MAVS signaling. Similarly, Swine fever virus induces mitochondrial fission and mitophagy to enhance apoptosis (30). By inducing mitophagy, viruses such as HCV or HBV not only interfere with anti-viral response but also attenuate mitochondria-induced apoptosis, which favors viral replication and persistent infection (31, 32).

Beyond its primary function to recognize and selectively remove damaged mitochondria, mitophagy thus influence various physiological processes, and it is not surprising that mitochondrial dysfunction is associated with many pathological conditions (Figure 1). The next chapter details the molecular mechanisms and the factors responsible for the induction of mitophagy with a particular focus on Optn, before presenting the pathologies associated with mitophagy.

## Molecular Mechanics of Mitophagy Induction

**Figure 2:**
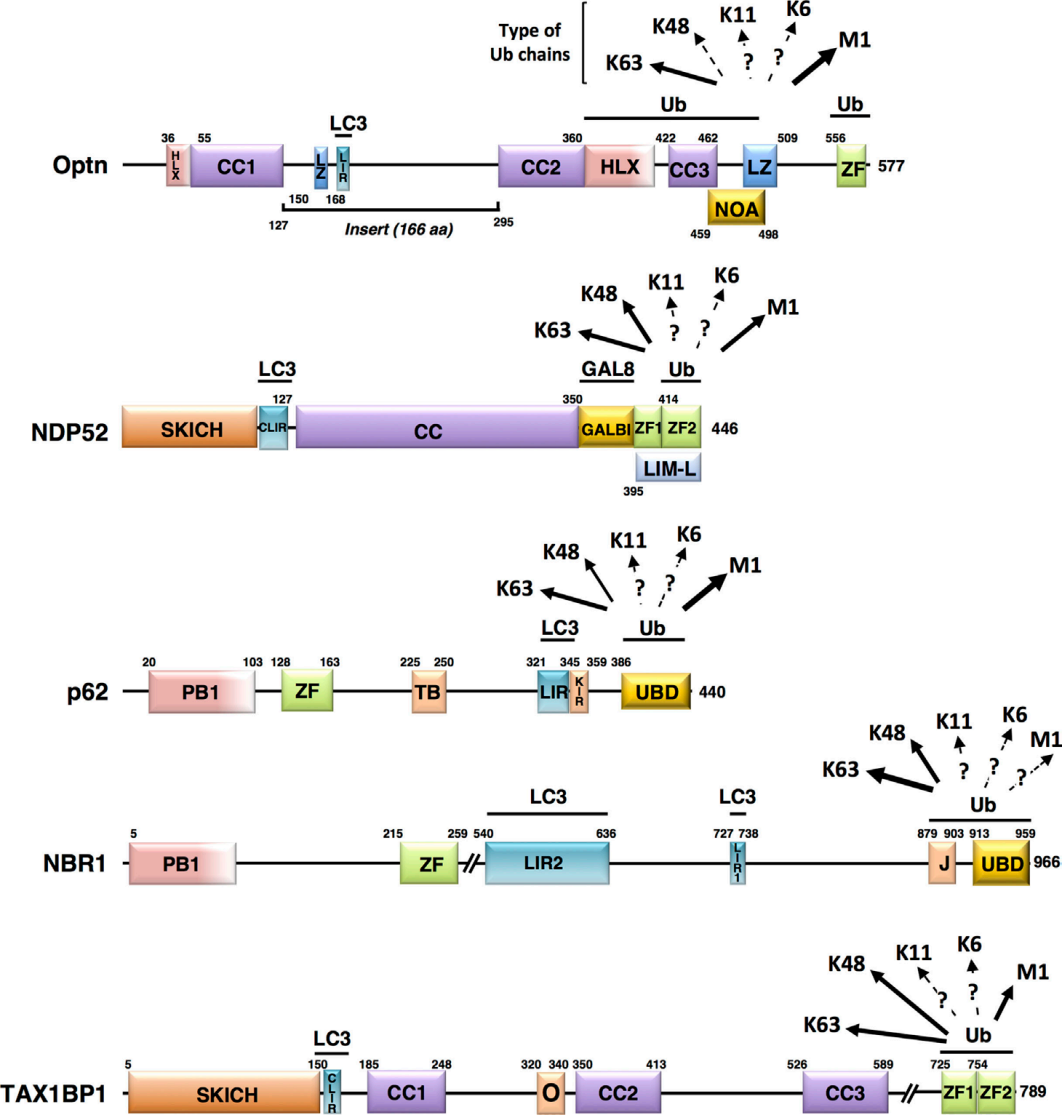
Schematic representation of the structural domains of the non-mitochondrial autophagy receptors involved in mitophagy. Each domain is represented, scaled according to its aa position [except for NBR1 and Tax1 binding protein 1 (TAX1BP1)] and delimitations are indicated. Abbreviations: HLX, helix-loop-helix; CC, coiled-coil; LZ, leucine zipper; ZF, zinc finger; LIR, LC3-interacting region; NOA, NEMO-optineurin (Optn)-ABIN domain; SKICH, SKIP carboxyl homology domain; CLIR, non-canonical LC3-interacting region; GALBI, galectin-8 binding region; LIM-L, Lin11, Isl-1, and Mec-3 (LIM)-like domain; PB, Phox and Bemp1 domain; TB, TRAF6-binding domain; KIR, Keap1-interacting region; UBD, ubiquitin-binding domain; J, α-helical J domain; O, homodimerization domain. Known preferential affinities of the autophagy receptors for the different ubiquitin chain types are presented according to the following references: Optn (37, 38); nuclear dot protein 52 (39); p62 (40, 41); NBR1 (42, 43), and TAX1BP1 (44, 45).

Mitophagy is the autophagy area in which most important advances has been made over the past 10 years, particularly regarding the knowledge of the molecular mechanisms of induction. All the different types of mitophagy require a receptor-mediated mechanism that promotes physical connections between the mitochondria and the autophagosomal membrane (33). These receptors can either be proteins or lipids localized to the mitochondrial membrane, but also non-mitochondrial proteins that interact both with ubiquitin-labeled mitochondrial surface and with the nascent autophagosome structure (34). Concomitant to the engulfment of ubiquitin-labeled mitochondria, conjugation of the cytosolic form of LC3, called LC3-I to phosphatidylethanolamine (PE) generates a LC3-PE conjugate (LC3-II), which is recruited to the isolated membrane. Partial mitochondrial degradation can also occur by the formation of mitochondrial-derived vesicles (MDVs) that can subsequently fuse with lysosomes. In addition, mitochondrial depolarization can lead to degradation of several outer mitochondrial membrane (OMM) proteins by the 26S proteasome (UPS process). Internally, degradation occurs *via* the mitochondrial unfolded protein response (UPR^mt^), which involves proteases. Optn (“Optic neuropathy inducing” protein, Optn) has been identified as a cytosolic receptor critically involved in the elimination of damaged mitochondria (35), and we will, therefore, focus our attention on the mitophagy pathway mediated by non-mitochondrial anchored autophagy receptors. The best-studied pathway mediated by these receptors involves the PTEN-induced putative kinase 1 (PINK1) and the E3 ubiquitin ligase Parkin, involved in familial Parkinson’s disease (36). Activation of these proteins leads to recruitment of the autophagy receptors that contain both the LC3-interacting region (LIR) motif binding the LC3-II form, and one or several ubiquitin-binding domain (UBD) interacting with ubiquitinated proteins on the targeted mitochondria (Figure 2). In the next section, the characteristics of the mitophagic receptors that have been identified to date will be presented.

### Mitophagic Receptors

Induction steps of mitophagy require the independent recruitment of the serine/threonine kinase, ULK1, the membrane spanning Atg9 protein, and LC3 to mitochondria. The mechanism by which these components are recruited to autophagosome-forming membranes growing around mitochondria involves autophagic receptors, such as p62/SQSTM1, neighbor of BRCA1 gene 1 (NBR1), nuclear dot protein 52 kDa (NDP52), Tax1 binding protein 1 (Tax1BP1), and Optn, as presented in Figure 2. Mechanistically, autophagy receptors are not recruited to mitochondria in the absence of damage, although constitutive ubiquitination is present at the outer mitochondrial membrane. In fact, the triggering signal is thought to rely on the activation of the E3 ubiquitin ligase Parkin that increases local concentration of ubiquitin to promote autophagy receptor recruitment. Although these receptors appear functionally redundant in mitophagy, Narendra et al. (46) observed that p62-KO cells can still degrade mitochondria through the autophagic machinery, and two studies found that primary PINK1/Parkin-dependent mitophagic receptors include Optn and NDP52, while p62 and NBR1 are not essential (35, 47). Using pentaKO HeLa cells in which all five endogenous receptors (p62, NBR1, NDP52, Tax1BP1, and Optn) were depleted by the CRISPR/Cas9 approach, it was observed that all re-expressed receptors are recruited to damaged mitochondria, but only re-expression of Optn and NDP52 (and TAX1BP1 to a lesser extent) could rescue mitophagy as measured by COXII and mtDNA degradation (35).

#### Optn and NDP52

The involvement of Optn and NDP52 in Parkin-dependent mitophagy was emphasized by the following observations: mutation of the UBD of Optn and NDP52 prevents their mitochondrial recruitment, and artificial targeting of PINK1 to the OMM of undamaged mitochondria in Parkin-depleted cells was sufficient to promote recruitment of both proteins, as well as a low level of mitophagy (35). Accordingly, Moore et al. (48) reported that Optn, NDP52, as well as TAX1BP1, are recruited to mitochondria with similar kinetics following either mitochondrial depolarization or localized generation of ROS leading to sequestration by the autophagosome.

Optineurin is a 67 kDa protein, also named FIP-2 (E3-14.7K-interacting protein), TFIIIA-INTP (transcription factor IIIA-interacting protein), HYPL [Huntingtin (Htt) yeast partner L], HIP7 (Htt-interacting protein 7), and NRP (NEMO-related protein) (49). Optn primary sequence and domain organization exhibit high homologies with NEMO (NF-κB essential modulator). Similar to NEMO, Optn can form oligomers following specific stimulation, such as oxidative stress, and can be found in high molecular complexes with various interacting proteins (50, 51). However, Optn diverges from NEMO (53% similarity in amino acid primary sequences) by the presence of an “insert” region of 166 amino acids located in the amino-terminal region. This region contains a leucine-zipper domain, a LIR that binds GABARAP and LC3 family members, and a binding domain for the small GTPase Rab8, a regulator of membrane trafficking. Similar to NEMO, Optn includes two coiled-coils, a UBD named NOA (NEMO-Optn-ABIN) and a C-terminal ubiquitin-binding zinc finger (ZF) depicted in Figure 2. Except for clearance of protein aggregates, ubiquitin-binding activity of Optn is required for its function in xenophagy and mitophagy. Optn is expressed in most cells and tissues, and its expression can be induced by TNF-α and interferons, probably as a result of NF-κB activation by these cytokines, since Optn gene possesses a functional NF-κB binding site in its promoter (50, 52). In contrast to NEMO, Optn is involved in several biological functions that are not related to NF-κB, including signaling pathways and host defense mechanisms [reviewed in Ref. (53)]. Wong and Holzbaur (54) first demonstrated the involvement of Optn in mitophagy *via* its active recruitment to Parkin-positive mitochondria and stabilization by its UBD. TANK-binding kinase-1 (TBK1) acts as an upstream regulator of mitophagy by phosphorylating Optn at multiple sites (see below) to enhance its interaction with LC3 and promote recruitment to ubiquitin-labeled mitochondria.

Most of the data on the autophagic functions of NDP52/CALCOCO2 comes from studies on the macroautophagy of intracellular pathogens. NDP52 structure includes: a N-terminal SKIP carboxyl homology domain (SKICH) responsible for its interaction with a TBK1 binding adaptor (AZI2/NAP1), and a noncanonical LIR motif that mediates its interaction with LC3, as shown in Figure 2 (55, 56). NDP52 can dimerize and associate with the E3 ubiquitin ligase leucine-rich repeat and sterile a motif containing 1 (LRSAM1) through a coiled-coil domain, located on its intermediate region (57). C-terminal region of NDP52 contains a LGALS8/galectin8-interacting region and a zinc-finger domain that binds to ubiquitin-coated pathogens (55). The molecular mechanisms responsible for the interaction between NDP52 and LGALS8 have been recently characterized (58, 59). However, the selective recognition of ubiquitin by NDP52 is still unclear. Interestingly, both NDP52 and p62 contain a LIR motif and a UBD domain, but they are independently recruited to ubiquitin-coated bacteria, likely due to distinct ubiquitin chain-type binding specificities (60, 61). In fact, a recent study addressing the molecular basis of ubiquitin recognition of NDP52 revealed the presence of two ZF domains: a dynamic unconventional ZF1 and a canonical C2H2-type ZF2 (39). However, only ZF2 can specifically bind to mono-ubiquitin or to K48-linked, K63-linked, and M1-linked polyubiquitin chains.

In addition to their autophagy receptor role, Optn and NDP52 are also involved in other steps of mitophagy. Mitophagy initiation involves the independent recruitment of ULK1 and Atg9 proteins, followed by the VPS34 lipid kinase complex generating phosphatidylinositol 3-phosphate that allows recruitment of double FYVE domain-containing protein 1 (DFCP1) and WD repeat domain phosphoinositine-interacting protein 1 (WIPI1). Surprisingly, knockout cells lacking both Optn and NDP52 (as well as the penta-KO cell line that lacks the five mitophagic receptors) failed to form the DFCP1- and WIPI1-coated isolation membrane on mitochondria and to recruit ULK1 for autophagosome initiation (35). This observation fits with a model in which preformed isolation membranes are not recruited to mitochondria, but are built at the OMM (62). ULK1 and DFCP1 recruitment and mitophagy could be rescued in the pentaKO cells with Optn or NDP52, but not with p62, indicating the unique ability of Optn and NDP52 to initiate autophagosome formation through ULK1, at least in mitophagy (35). Despite functionally redundant action, Optn and NDP52 display distinct tissue distributions, the first being highly expressed in brain while the second is below detection in these cells (35), suggesting that they may function differentially according to tissue specificity. Further analyses of midbrain of normal rats indicated an enriched expression of Optn in dopamine neurons (63). While efforts are being concentrated to elucidate the distinct roles of the mitophagy receptors, several interplays between these adaptors have been reported. For example, Liu et al. (64) found that ubiquitination of Lys193 of Optn by the ubiquitin ligase HACE1 promotes its interaction with p62 and increases the autophagic flux. Redundancies between mitophagy receptor functions have also been proposed, since Optn and NDP52 are located on common subdomains of ubiquitinated *Salmonella*, while p62 and Optn localize to disconnected subdomains (37).

#### p62/Sqstm1, NBR1, and TAX1BP1

Although not playing a critical role, p62, NBR1, and TAX1BP1 also participate to mitophagy (35). p62/SQSMT1 was also detected in ubiquitinated protein aggregates (65, 66). In addition to its C-terminal UBD and LIR sequence (Figure 2), p62 possesses a PB1 domain responsible for its heteromerization and interaction with other proteins such as NBR1 (42). Knockout studies of p62 in mice and *Drosophila* showed that this receptor is involved in aggregation and autophagic clearance of ubiquitinated proteins (67, 68). p62 can also deliver ubiquitinated cargos to the proteasome (69). By shuttling between the nucleus and the cytoplasm, p62 can deliver nuclear ubiquitinated substrates to the autophagy pathway (65). In fact, aggregates containing p62 and ubiquitinated proteins may serve as a nucleating scaffold for autophagosome biogenesis (70). However, the role of p62 in autophagy induction appears to be complex and context-dependent (71). p62 can inhibit autophagy by promoting mTORC1 activation that phosphorylates ULK1/2, but it can also liberate Beclin1 by disrupting its association with Bcl-2 and thus induce autophagy (72). Remarkably, cytoplasmic p62 levels serve as an autophagy indicator—i.e., its amount is inversely correlated with autophagic activity—since cytosolic p62 is itself degraded by autophagy (73).

NBR1 not only participates in the recruitment and autophagosomal degradation of ubiquitinated proteins through its UBD and LIR (Figure 2) but it can also interact with p62 *via* its own PB1 domain (74). Similar to p62, NBR1 contains a Zinc-finger domain, but includes a J domain as well as two LIR sequences, although LIR2 does not have the core consensus motif W/YXXL/I LC3-interacting sequence [reviewed in Ref. (33)]. Due to their high similarities, NBR1 and p62 receptors were suggested to act cooperatively to target polyubiquitinated aggregates and even whole organelles to degradation (43). However, NBR1 displays higher affinity for ubiquitin than p62, likely due to concomitant binding of the J domain and UBD (42).

Tax1 binding protein 1 was originally identified as a protein interacting with the human T-cell leukemia virus 1 Tax protein, the NF-κB regulatory proteins A20 and TRAF6 (75). TAX1BP1 serves as an adaptor molecule that recruits the E3 ligases Itch and RNF11 to A20 to terminate NF-κB signaling and proinflammatory cytokine production (76, 77). TAX1BP1 also cooperates with A20 to restrict RIG-I/MDA5-mediated signaling and the induction of IFN-β during RNA virus infection by inhibiting the K63-linked polyubiquitination of TBK1 and its homolog IKKε (78). As for Optn and NDP52, TAX1BP1 also acts as an ubiquitin-binding autophagy receptor in both clearance of *Salmonella* and the mitophagy process (44, 79).

#### Additional Adaptors

In addition to these receptors, other adaptors also act on removal of damaged or excessive mitochondria (such as Atg32 in yeast and NIX in mammals) by recognizing proteins at the surface of mitochondria and ensuring their delivery to the maturing autophagosome by their LIR sequence (80, 81). NIX/BNIP3L (BCL2/adenovirus E1B 19 kDa interacting protein 3-like), a member of the Bcl-2 family, is a mitochondrial outer membrane protein, involved in the elimination of erythrocyte mitochondria, which can bind to LC3 through its LIR sequence (82). NIX was also shown to promote clearance of damaged mitochondria after cell treatment with mitophagy inducers, although mutation of its LIR domain has only a partial effect *in vivo* (83).

### Regulation of the Mitophagy Process

**Figure 3:**
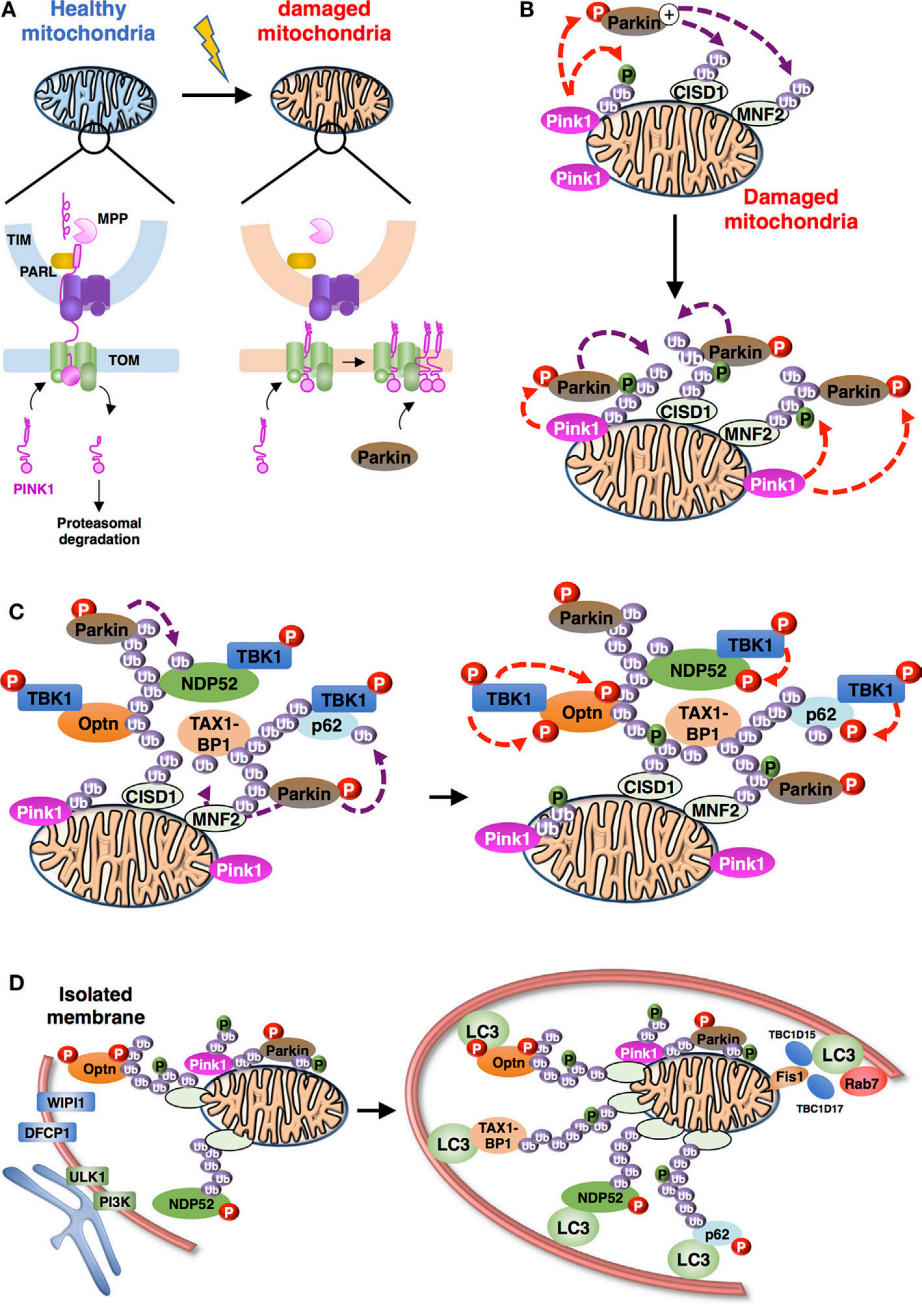
Schematic representation of the overall process of mitophagy [adapted from Ref. (36)]. **(A)** PINK1 import and accumulation mechanisms into mitochondria. PINK1 detects mitochondrial damage *via* selective proteolysis. Continuous import and the degradation cycle explain the undetectable levels of PINK1 on healthy mitochondria. Upon damage, import into the inner membrane is blocked leading to accumulation of uncleaved PINK1 on the outer mitochondrial membrane. Abbreviations: TOM, translocase of the outer mitochondrial membrane complex; TIM, translocase of the inner mitochondrial membrane complex; MPP, mitochondrial processing peptidase; PARL: presenilin-associated rhomboid-like protein. **(B)** Positive feedback ubiquitination cycles induced by Parkin and Ub chains on damaged mitochondria. Mitochondria-recruited PINK1 phosphorylate Ub chains conjugated to mitochondrial proteins, including CISD1 and MNF2 (shown here), as well as cytosolic Parkin, subsequently recruited to mitochondria. Activated Parkin can elongate Ub chains that can be, in turn, phosphorylated by PINK1. **(C)** Recruitment and activation of autophagy receptors during Parkin/PINK1-mediated mitophagy. Autophagy proteins, including adaptors [Optineurin (Optn), nuclear dot protein 52 (NDP52), p62, and Tax1 binding protein 1 (TAX1BP1) presented] and regulators (TANK-binding kinase-1, TBK1) are recruited to the mitochondria. TBK1-phosphorylation of autophagy adaptors can enhance their Ub-binding activities and promote the recruitment of the light chain 3 (LC3)-labeled isolation membrane. **(D)** Autophagosome biogenesis during mitophagy. Formation of autophagophore vesicle surrounding damaged mitochondria is a multi-independent process. (i) Optn and NDP52 mediate initiation and elongation of autophagosomes (at ER-mitochondria contact sites for example) *via* recruitment of complexes containing ULK1, PI3K, WIPI1, and DFCP1. (ii) Autophagy receptors also mediate recruitment of LC3/GABARAP family members to promote expansion of the isolation membrane. (iii) Finally, Rab-GAPs, TBC1D15, and TBC1D17, localized to mitochondria *via* interaction of Fis1 can regulate proper autophagosomal formation by modulating Rab7 activity.

Recruitment of cytosolic receptors to damaged mitochondria through their UBD and their interaction with LC3 family members constitute the two main functions of these adaptors that critically link the upstream induction cascades to autophagosome formation. Several regulatory mechanisms take place to ensure adequate and efficient control of these temporally and spatially controlled functions. Some of these recently described molecular mechanisms are depicted in the following paragraphs and illustrated in Figure 3.

#### TBK1 as a Regulator of Mitophagy

TANK-binding kinase-1 is a serine/threonine kinase involved in intracellular pathways including innate immune response, xenophagy, cell growth and proliferation (84–86). As for the members of the IκB kinase (IKK) family, TBK1 is activated by phosphorylation on its activation loop (serine 172). TBK1 is maintained in an inactive form until adaptor proteins recruit it to signaling complexes where it can autophosphorylate or be phosphorylated by other kinases, indicating that localization of TBK1 is critical for its activation (87, 88). Specificity is achieved through the binding of TBK1 to unique adaptors that connect it to distinct signaling complexes (89). During xenophagy, TBK1 phosphorylates Optn at S177 to promote its interaction with LC3 and GABARAP family members and ensure bacterial elimination (37). S177 phosphorylation of Optn was further shown to be required for clearance of aggregates and mitophagy (35, 47, 90). Structural analyses showed that the side chain of Arg11 and Lys51 in LC3B recognizes the negative charge induced by phosphorylation (70). Phosphorylation might be a general mechanism of regulation of selective autophagy, since many autophagy receptors, including NIX and NBR1, contain conserved serine residues adjacent to their LIR sequence. Mitophagy induction was shown to stimulate TBK1 kinase activity and, more specifically, its autophosphorylation at S172. Proteomic analyses demonstrated that, after PINK1/Parkin engagement, TBK1 phosphorylates Optn on two additional sites, S473 and S513 and that these modifications enhance its binding to ubiquitin and its retention on damaged mitochondria (91). Strikingly, S473 phosphorylation by TBK1 not only increases affinity of Optn toward all ubiquitin chain types but it also enables Optn to bind to phosphorylated S65 ubiquitin (see paragraph on PINK1 below), while recombinant Optn did not bind efficiently pS65-Ub *in vitro*. TBK1 also phosphorylates NDP52, p62, and TAX1BP1 on multiple autophagy-relevant sites, including the UBD of p62, SKICH domains of NDP52, and TAX1BP1 and LIR domains of p62 [Figure 2 (36, 91, 92)]. Despite phosphorylation on multiple autophagy receptors, controversial data were obtained regarding the molecular mechanism by which TBK1 plays its role in mitophagy; one group reported that mitophagy was inhibited by the TBK1 knockout, while it was found by others that only the combined TBK1 and NDP52 deficiency could impair it (35, 47). Nevertheless, both studies acknowledge the critical role of TBK1 kinase activity in mitophagy in agreement with the fact that TBK1 is recruited together with Optn to depolarized damaged mitochondria (79).

#### LC3/GABARAP Family Members

Light chain 3/GABARAP family members have distinct roles in the mitophagy process, as shown by the observation that cells lacking Atg3, a component of the LC3/GABARAP lipidation machinery, did not properly engulf mitochondria into autophagosomes and failed to seal the autophagosomal membranes (62). Accordingly, EM images of Parkin-mediated mitophagy reveal the presence of LC3-positive membrane in restricted mitochondria regions. At late stages of mitophagy, LC3/GABARAP family members are thought to promote expansion and sealing of the autophagosome. Two mitochondrial localized Rab-GTPase-activating proteins, TBC1D15 and TBC1D17, control the isolation membrane (cup shaped membrane or phagophore, which elongates and closes to form a mature autophagosome) engulfment of mitochondria by binding to the OMM protein Fis1 (93). This function is achieved through interaction with LC3/GABARAP members and is regulated by Rab7, a late endosome-/lysosome-associated small GTPase. Mitochondrial engulfment to LC3-labeled autophagosomes is, therefore, mediated by both the autophagic receptors and the Rab7-associated TBC1D15/17 pathway (Figure 3), although potential links between these components remain to be explored. Interestingly, autophagic receptor penta-KO cells displayed efficient LC3 lipidation despite their inability to form mitochondria-associated autophagosomes, suggesting independent regulatory mechanisms of LC3 processing and autophagosomal membrane biogenesis (35).

### PINK1 and Parkin

As mentioned before, autophagy receptors are recruited to dysfunctional mitochondria following identification and labeling of these organelles by ubiquitin. Sensing mitochondrial damage is achieved, at least for the major part, by the PINK1/Parkin pathway. Interestingly, relationships between mitochondrial dynamics and pathogenesis have gained intense interest following the discovery that two Parkinson’s disease genes, *PINK1* and *Parkin*, also regulate mitophagy. Although a causative role of autophagy receptors in mitophagy-associated pathologies including neurodegenerative diseases has yet to be defined, we will first describe the molecular mechanisms of PINK1/Parkin-induced mitophagy. We will then explore the potential links between components of the mitophagy and pathologies.

#### PINK1, a Sensor of Mitochondrial Damage

PINK1 is a mitochondria-localized kinase, which is, under normal steady state conditions, imported through the translocase of the outer mitochondrial membrane (TOM) complex and into the translocase of the inner mitochondrial membrane (TIM) complex, where it is cleaved by the mitochondrial processing peptidase (Figure 3A). Then, the presenilin-associated rhomboid-like protein (PARL), an inner mitochondrial membrane protease, cleaves PINK1, generating a 52 kDa, N-terminal deleted form of PINK1 further degraded by the ubiquitin proteasome system. Depolarization of the mitochondrial membrane potential that regulates protein import into mitochondria, results in PINK1 accumulation on the outer membrane (46, 94). The accumulated PINK1 phosphorylates the E3 ligase Parkin, which is subsequently activated and recruited to mitochondria (95–99). In detail, stabilization of PINK1 at the outer membrane allows formation of a large complex (around 700 kDa) including TOM machinery and at least a dimer of PINK1 that is activated through its autophosphorylation on S228 and S402 residues (100–103). PINK1 directly phosphorylates the S65 residue of the UBD of Parkin and stimulates its E3 ligase activity and recruitment to mitochondria (104, 105). However, S65-mutated or UBD-depleted Parkin is still recruited to mitochondria with a PINK1 kinase-dependent mechanism suggesting that a cytosolic substrate of PINK1 is involved in Parkin activation and recruitment (106). Three independent groups reported that PINK1-mediated phosphorylation of ubiquitin at S65 (thereafter called pS65-Ub) plays a critical role in Parkin activation and mitophagy (107, 108). Strikingly, this region of ubiquitin is highly homologous to the UBD of Parkin that contains the sequence of phosphorylation by PINK1. In fact, ubiquitin chains that are attached to the outer membrane proteins can be phosphorylated by PINK1 and may serve to recruit Parkin on mitochondria (109). Ordureau et al. further show that phosphorylated Parkin binds to pS65-Ub with a higher affinity than its unmodified form, suggesting that Parkin is first phosphorylated by PINK1 and then further activated by pS65-Ub. Mitochondrial recruitment and activation of Parkin are initiated by pS65-Ub moieties that are linked to OMM proteins such as mitofusin 1 (Mfn1), indicating that these phosphorylated ubiquitins act as an allosteric effector of Parkin ubiquitin ligase activity. Furthermore, the mitochondrial recruitment of Optn and NDP52 is prevented by expression of the kinase-dead form of PINK1. Consistently, PINK1-mediated ubiquitin phosphorylation enhances recognition of pS65-Ub by NDP52 and Optn (35). In contrast, p62 exhibits no difference in its binding affinity to Ub and pS65-Ub and its recruitment to mitochondria seems independent of phosphorylated ubiquitin chains. Other mitochondrial substrates of PINK1 have been identified: phosphorylation of Miro1 at S156 allows its Parkin-mediated proteasomal degradation to arrest mitochondrial motility (110). Mitofusin2 is also phosphorylated at T111 and S442 by PINK1 to induce its binding to Parkin, ubiquitination, and degradation, but is not involved in Parkin recruitment to mitochondria (111, 112).

#### Parkin E3 Ubiquitin Ligase

Parkin is an E3 ubiquitin ligase from RBR (ring between ring) domain-containing family of proteins that shares three tandem zinc coordination domains (113). As for HOIP (HOIL1-interacting protein, the catalytic component of the linear ubiquitin chain assembly complex LUBAC), Parkin discharges ubiquitin from an E2 onto a catalytic cysteine, yielding a thioester intermediate, before conjugating the ubiquitin onto a substrate (114–116). This allows RBR ligases to dictate the ubiquitin chain linkage generated, independently of the E2 used. Parkin contains four zinc-coordinating domains (RING0, RING1, IBR, and RING2), although only RING1 adopts the canonical ubiquitin ligase RING domain structure, and several E2s can transfer ubiquitin to PINK1-activated Parkin (117, 118). In untreated cells, cytosolic Parkin remains inactive in an auto-inhibitory conformation that blocks the E2 binding site in RING1 and masks the catalytic site (C431) in RING2 (119–121). Once bound to Parkin, pS65-Ub releases the auto-inhibitory interactions leading to an “open” conformation of Parkin that is further stabilized by its phosphorylation by PINK1 at S65 (Figure 3B) (122–124). Binding to phosphorylated ubiquitin may also have additional roles in mitophagy, i.e., association with proteasomal components (108, 125). Interestingly, interaction between Parkin and pS65-Ub does not require its catalytic residue; however, it is inhibited by L283P, G284R, and C352G pathogenic mutations that are located on the IBR and RING1 domains (109, 126). The ability of Parkin to bind to pS65-Ub constitutes a positive feedback model (Figure 3B): on mitochondria, Parkin generates Ub chains that are subsequently phosphorylated by PINK1 and serve as a docking site for Parkin to amplify labeling of damaged mitochondria. This mechanism could explain the robust Parkin translocation observed after mitochondrial membrane depolarization characterized by the almost complete depletion of cytosolic Parkin, even when overexpressed. Interestingly, pS65-Ub confers resistance to deubiquitinases (DUBs) and consequently enhances mitophagy (123, 127). Activation of Parkin leads to extensive ubiquitination of the mitochondrial outer-membrane proteins, some of which can be degraded by the 26S proteasome, these events being required for targeting of mitochondria to autophagic membranes (128, 129). The mitophagic process does not target healthy mitochondria as Parkin is selectively enriched at damaged organelles. Ubiquitin can be linked through any of its seven lysine residues (K6, K11, K27, K29, K33, K48, K63) or through the first Methionine (M1 or linear ubiquitination), yielding eight potential types of homogeneous polyubiquitin chains linked to various substrates. K48- and K11-linked polyubiquitin chains target proteins for destruction, while K63-linked chains allow the coordination of processes, such as endocytic trafficking, inflammation, translation, and DNA repair. The functions of other lysine-conjugated polyubiquitin chains have been less studied. Parkin modifies cytosolic and mitochondrial membrane proteins with K6- and K11-linked as well as K48- and K63-linked ubiquitin chains (128–130), although mitochondrial proteins displayed more K48- and K63-linked chains than K6 and K11 (109). Using K/R-mutated ubiquitin approach, Cunningham et al. (131) showed that mitophagy was impaired when either K6- or K11-linked chain formation were prevented. However, K6 chains generated by Parkin autoubiquitination reduced its ligase activity. This inhibitory effect is prevented by the USP8 DUB that removes the K6-linked ubiquitin chains present on Parkin, but not on mitochondrial proteins (132). Other DUB activities were shown to regulate mitophagy, including the mitochondria-anchored DUB USP30 that specifically cleaves Parkin-generated K6-, K11, and K63-Ub chains, globally antagonizing Parkin function (131). In addition, two other DUBs, namely USP15 and USP35 also regulate mitophagy. USP15 removes preferentially K48 and K63 chains on the mitochondrial surface, while the USP35 deubiquitinates cytosolic mitophagy factors, such as MFN2 and TOMM20, although the physiological roles of these DUBs in mitophagy remain to be fully investigated (133–135). Recent work by Szargel et al. showed that other E3 UB ligases including SIAH-1 catalyze the ubiquitination of OMM proteins, even in the absence of Parkin (136). Nevertheless, PINK1 is still required for the phosphorylation of ubiquitin, suggesting that pS65-Ub chains could be a signal for mitophagy receptors’ recruitment at the mitochondrial surface. Importantly, PINK1 and Parkin have also been implicated in an autophagy-independent degradation pathway, in which MDVs are carried to the late endosome network and fused to lysosomes (137, 138).

The primary role of K63-linked Ub chains is likely to recruit autophagy receptors to damaged mitochondria (96, 97, 139–141). Interestingly, mitochondrial K11- and K48-linked ubiquitin chains were shown to lead to an endoplasmic reticulum-associated degradation-like process of outer mitochondrial membrane proteins, required for mitophagy (128, 142, 143). Interestingly, Parkin is recruited to LUBAC under cellular stress, to increase linear ubiquitination of NEMO, suggesting that Parkin might be able to indirectly stimulate synthesis of linear ubiquitin chains on mitochondria (144). However, study employing fluorescent-based cellular sensors for ubiquitin chains indicated that Parkin-induced mitophagy predominantly involves K63-linked ubiquitin rather than M1-linked chains (140). Although the function of the distinct types of ubiquitin chains linked to mitochondria is yet unknown, it has been suggested that the mitophagic inducer may be the chain linkage type and density rather than the nature of Parkin substrates (129). This observation strongly argues for distinct functions of the autophagy receptors relative to their preferential binding activities toward specific type of ubiquitin chain linkage. Although some of current data on mitophagy receptor affinity for ubiquitin, summarized in Figure 2, are already known, extensive biochemical and structural studies are required to complete the overall picture.

## Optn-Mediated Mitophagy: Physiological and Pathological Aspects

Mutations of the *Optn* gene have been associated with several pathologies including neurological disorders, as well as with the development of normal tension glaucoma (NTG) [30% of primary open-angle glaucoma (POAG)] and POAG, one of the major causes of irreversible bilateral blindness. More recently, *Optn* mutations were found in patients with amyotrophic lateral sclerosis (ALS) and the *Optn* gene constitutes a risk factor for the development of Paget’s disease of bone [reviewed in Ref. (49)]. Mutations of Optn found in glaucoma and ALS patients are compiled in Table 1. ALS is characterized by the formation of aggregates composed of ubiquitinated proteins in affected motor neurons. These aggregates are predominantly caused by an accumulation of misfolded proteins resulting from mutations of the copper/zinc superoxide dismutase 1 (*SOD1*), the DNA/RNA binding protein TAR DNA-binding protein of 43 kDa (*TDP-43*), fused in sarcoma (*FUS*), ubiquilin-2 (*UBQLN2*) or C9ORF72, as well as from autophagic defects that may involve mutations of Optn, p62, or TBK1. These aggregations lead to formation of inclusions containing TDP-43 and optionally Optn (145, 146). A wider role of Optn in neurodegenerative pathologies has been suggested, since endogenous Optn is not only found in protein aggregates from ALS patients but also in other neurodegenerative diseases, such as Huntington’s, Alzheimer’s, Creutzfeld–Jakob’s, Parkinson’s, and Pick’s disease (147–149). Indeed, Optn has been detected in distinct types of ALS intraneuronal inclusions, in neurofibrillary tangles and dystrophic neurites in Alzheimer’s disease, in Lewy bodies and Lewy neurites in Parkinson’s disease, ballooned neurons in Creutzfeldt–Jakob’s disease, glial cytoplasmic inclusions in multiple system atrophy, and Pick bodies in Pick’s disease (149). The presence of these protein aggregates suggests that cellular clearance mechanisms such as autophagy (and possibly mitophagy) must be impaired in these diseases. In this part, we will discuss recent findings linking the involvement of the mitophagy function of Optn in these diseases, focusing mainly on neurodegenerative diseases.

**Table 1:** Gene mutations and nucleotide variations associated with open-angle glaucoma and amyotrophic lateral sclerosis (ALS).

Disease	Associated phenotype	Variant type	AA change	Nucleotide change	Exon or intron	Inheritance	Reference
POAG		Missense	H26D	c.76C>G	Exon	Het.	(150, 151)
		Coding-synonymous	T34T	c.102G>A	Exon	Homo.	(152, 153)
		Missense	E50K	c.148G>A	Exon	Het.	(151, 154)
		Missense	L54V	c.160C>G	Exon	Het.	(155)
		Missense	M98K	c.293T>A	Exon	Homo.	(153, 154)
		Missens	E103D	c.309G>C	Exon	Het.	(156)
		Missense	T202R	c.605C>G	Exon	Het.	(157)
		Missense	E322K	c.964G>A	Exon	Het.	(158)
		Missense	A336G	c.1007C>G	Exon	Het.	(159)
		Missense	A377T	c.1129G>A	Exon	Het.	(160)
		Missense	K435R	c.1304A>G	Exon	Het.	(161)
		Missense	H486R	c.1457A>G	Exon	Het.	(151, 156)
		Frameshift	D128Rfs*22	c.381_382insAG	Exon	Het.	(154)
		–	na	c.553-10G>A	Splice	Het.	(161)
		–	na	c.553-5C>T	Splice	Homo.	(161)
		–	na	c.626+24G>A	Splice	Het.	(156)
		–	na	c.1401+21C>G	Splice	Het.	(156)
	fALS	Comp.optineurin (Optn)	M98K+G291fs	na	Exon	Homo.	(162)
	fALS	Composite	M98K+E322K+TDP-43 (N352S)	na	Exon	Het.	(163)
	fALS	Missense	R545Q	c.1634G>A	Exon	Het.	(164)
	FTD	Deletion	127fs*21	691_692insAG	Exon	Both	(165)

sALS		Null	No protein	Δe1-4	Exon	Het.	(166)
		Null	No protein	Δe3-5	Exon	Het.	(166)
		Missense	H3Y	c.7C>T	Exon	Het.	(167)
		Missense	P16A	c.46C>G	Exon	Het.	(167)
		Missense	A136V	c.407C>T	Exon	Het.	(168)
		Missense	G159V	c.476G>T	Exon	Het.	(169)
		Missense	V161M	c.481G>A	Exon	Het.	(170)
		Deletion	p148_184del	c.552+1delG	Intron	Het.	(171)
		Missense	T282P	c.844A>C	Exon	Het.	(164)
		Missense	Q314L	c.941A>T	Exon	Het.	(164)
		Missense	K395R	c.1184A>G	Exon	Het.	(168)
		Deletion	na	1401+2T>G	Intron	Het.	(172)
		Deletion	na	c.1401+4A>G	Intron	Het.	(164)
		Missense	L494W	c.1481C>G	Exon	Het.	(173)
		Missense	E516Q	c.1546G>C	Exon	Het.	(163)
		Missense	L568S	c.1703T>C	Exon	Het.	(164)

fALS		Missense	K59N	c.177G>G	Exon	Het.	(174)
		Missense	R96L	c.287G>T	Exon	Het.	(165)
		Nonsens	S174X	na	Exon	Homo.	(175)
		Missense	V295F	c.883G>T	Exon	Het.	(176)
		Frameshift	K440Nf*8	c.1320delA	Exon	Het.	(163)
		Missense	M447R	c.1340T>C	Exon	na	(172)
		Missense	I451T	c.1352T>C	Exon	Het.	(168)
		Missense	Q454E	c.1360C>G	Exon	Het.	(169)
		Missense	A481V	c.1442C>T	Exon	Het.	(174)
		Missense	L500P	c.1499T>C	Exon	na	(177)
		Missense	K557T	c.1670A>C	Exon	Het.	(167)

ALS:f>s		Missense	Q398E	c.1192C>G	Exon	na	(178)

both		Frameshift	58aa or no protein	Δe5fs	Exon	Homo.	(166)
		Nonsens	Q165X	c.493C>T	Exon	Het.	(179)

ALS		Nonsens	G23X	c.67G>T	Exon	Het.	(164)
		Missense	A93P	c277G>C	Exon	Het.	(180)
		Comp. Optn only	D220Mfs*12+Q165X	na	Exon	Het.	(179)

FTD	fALS	Comp. Optn	Q235X+A481V	c.703C>T+c.1442C>T	Exon	Het.	(181)
	fALs	Frameshift	359fs*	na	Exon	Homo.	(162)
	sALS	Nonsens	Q398X	c.1502C>T	Exon	Homo.	(182)
	sALS	Missense	E478G	c.1433A>G	Exon	Both	(183)
		Composite	G538Efr*27+TANK-binding kinase-1 (R177X)	c.1243-740-740_1612+1292delins25+c.349C>T	Exon	na	(181)

*Mutations or nucleotide variations of human Optineurin are listed according to the reported phenotype (POAG, primary open-angle glaucoma; s and fASL, sporadic and familial ALS; FTD, frontotemporal dementia). Asterisks denote stop codons with the following number corresponding to the preceding codon*.

### Involvement of Optn Mitophagic Function in POAG

**Figure 4:**
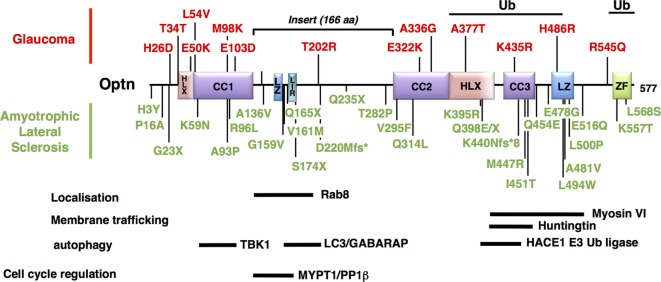
Mapping of the neurodegenerative disease-associated mutations of Optineurin (Optn). Localization of the disease-associated polymorphisms and mutations of human Optn protein is shown (as described in Table 1). Mutations associated with POAG and juvenile open-angle glaucoma are indicated in red, while mutations linked to amyotrophic lateral sclerosis are in green. Optn contains multiple protein interaction domains that are important for its localization and its mitophagy-related function. The regions of human Optn that have been involved in the interaction with its different partners are represented as thick bars with their delimitations.

Glaucoma is one of the most important worldwide disease responsible for blindness, and caused by gradual loss of RGCs (184). Its most common form is POAG. Pathogenesis has been partially linked to mutations of genes that include *myocilin, WD40-repeat36, Neurotrophin 4*, and *Optn* (185). The most frequent *Optn* mutation, E50K, displays the strongest genetic linkage with POAG, while H486R is associated with juvenile open-angle glaucoma (JOAG), a more severe and rare form (186). Subsequently, *Optn* mutations have been associated with both familial and sporadic forms of normal tension glaucoma (NTG, a subgroup of POAG) (187, 188). Most of the *Optn* gene alterations observed is missense mutation in a single copy, suggesting dominant phenotypes (Table 1). The mechanism by which these mutations induce glaucoma is unclear, but unlike other mutants, such as H486R, H26D, T202R, the expression of E50K and M98K (Figure 4) in RGC have been clearly shown to induce cell death compared to wild-type Optn (189, 190). Degenerated RGCs and narrowed retinal cell layers were thus observed in transgenic mice expressing E50K-Optn (191). As Rapamycin, an autophagy inducer, protects cells from E50K-Optn induced death, loss of RGC in transgenic mice was suggested to involve inhibition of autophagy (192, 193). Indeed, increased LC3 and p62 levels, enlargement of LC3-positive vesicles and inhibited autophagy are observed in E50K-Optn expressing cells. The mechanism by which E50K-Optn inhibits autophagy involves its interaction with TBC1D17, a Rab-GTPase-activating protein for Rab8, which inhibits autophagy through its catalytic activity. As mentioned before TBC1D17 also plays a role in the regulation of engulfment of mitochondria to the isolation membrane of nascent autophagosomes, suggesting a yet uncovered link between E50K-Optn mutant and defaults in mitophagy. Such a link was recently strengthened by the finding that E50K-Optn induces mitochondrial fission-mediated mitophagy in the axons of the glial lamina of aged E50K transgenic mice (194). The role of autophagy inhibition in ROS production by E50K-Optn was also investigated. Overexpression of E50K-Optn in retinal cells alters ROS production, inhibition of which prevents E50K-Optn-induced cell death (190). It was later shown that the oxidative stress induces the formation of covalent trimers of E50K-Optn that is prevented by antioxidants and could be involved in glaucoma pathogenesis (51). Finally, it was reported that overexpression of M98K-Optn induces autophagy, which leads to the degradation of cellular transferrin receptor protein (TFRC), and causes cell death selectively in retinal cells (189). This process is dependent upon M98K-Optn interaction with ubiquitin chains, LC3 and Rab12. In conclusion, a role for Optn in macroautophagy and mitophagy in retinal cells is clearly established and these cellular processes certainly contribute to RGC death in glaucoma. However, the molecular mechanisms by which the M98K or E50K-Optn mutant enhances and blocks autophagy, respectively, are not clear. The Optn effector TBK1 is also a glaucoma-associated protein involved in macroautophagy and mitophagy, and shows increased interaction with E50K-Optn. Thus, early-onset familial NTG with autosomal dominant inheritance is related to copy number variations of TBK1 (195, 196). *Optn* and *TBK1* mutations account for 2–3% of NTG and patients carrying these mutations develop severe disease prior to 40 years old. Future work will determine how these two proteins interact functionally in retinal cells.

### Dynamics of Mitochondrial Degradation in Neurodegenerative Diseases

#### Mutations of Autophagy/Mitophagy Components in ALS

Amyotrophic lateral sclerosis is a rare neurodegenerative disease characterized by the death of motor neurons in the spinal cord that control voluntary muscle movements. Unable to function the muscles progressively weaken and become atrophic. At the ultimate phase, the patients are unable to speak, eat, or breathe. Most ALS cases are sporadic (sALS) and only 10% of cases are inherited forms (fALS). These genetic forms have made it possible to highlight factors involved in the disease etiology, such as the *SOD1* gene that accounts for around 20% of familial ALS cases, TDP-43 and FUS, as well as p97/valosin-containing protein (VCP), UBQLN2, p62/SQSTM1, and Optn (145, 146, 197–199). As many recent reviews already evaluated the role of autophagy related proteins in ALS [see for example, Ref. (34, 200, 201)], only data related to Optn will be presented here. The link between *Optn* and ALS is very strong, since almost 40 variants of *Optn* gene have been described in ALS to date (Table 1) (147, 169, 179, 202). The pathogenicity of ALS-linked *Optn* mutations has been shown. Optn causes the disease by protein insufficiency or loss-of-function and, thus, native Optn form is thought to exert a protective role. Accordingly, upregulation of Optn was observed in ALS cases caused by mutations in other ALS-associated genes, suggesting that Optn may mediate protective and/or compensatory functions during neurodegeneration.

#### Pathological Mutations of Optn and the Mechanism of ALS

Optineurin mutations are more frequent in Japanese and Chinese patients (3% fALS) compared to the Caucasian population (<1% fALS), although a great heterogeneity of mutations has been observed (Table 1). Complete deletions of the *Optn* gene have been reported, resulting from the loss of the initiator codon in exon 4 and the deletion of exon 5 (180). Nonsense or frameshift mutations in homozygous or heterozygous states (suggesting that haploinsufficiency can cause pathology) have also been reported, resulting in non-functional altered Optn proteins (see Table 1 for reference). Interestingly, about 30 missense loss-of-function mutations have been found, mainly in the C-terminal ubiquitin binding or oligomerization domain, suggesting the importance of these regions in the pathogenicity of ALS. Importantly, mutations in Optn (E478G and Q398X) that disrupt ubiquitin binding and affect mitophagy have been linked to ALS (36). TBK1 mutations have also been identified to cause ALS, while TBK1 gene duplications have been linked with glaucoma (195, 203). It is, therefore, tempting to speculate that defects in mitophagy are involved in the development of both diseases in agreement with the presence of damaged mitochondria (204, 205). However, neurodegeneration in ALS and glaucoma have also been linked to other TBK1- and Optn-related biological processes, including xenophagy (37, 55), inflammation (206, 207), and innate immunity [reviewed in Ref. (86)]. In addition, these functions may partially overlap [see for example, Ref. (208)], illustrating the difficulty of establishing the contribution of TBK1 and Optn in these neurodegenerative diseases. Optn mutations have been also found in association with TBK1, TDP-43, or C9ORF72 mutations in ALS (163, 209). As for SOD1, VCP, UBQLN2, p62, or TBK1, loss-of-function mutations of Optn have also been implicated in FTD (162, 181, 183).

Due to the pleiotropic functions of Optn, these mutations could be involved in ALS by interfering with several biological processes linked to autophagy/mitophagy. (1) Lysosomes trafficking: as Optn interacts with Htt, the protein mutated in the neurodegenerative disorder Huntington’s disease (210). Htt is critical for the regulation of post-Golgi trafficking to the plasma membrane and to lysosomes (211, 212). Consequently, Htt deficiency alters the spatial organization of late endosomes/lysosomes. Interestingly, abnormal polyglutamine expansion (mHtt) results in Optn and Rab8 delocalization from the Golgi apparatus and, in turn, alters the post-Golgi trafficking to the lysosomes, suggesting that this function of Htt is dependent on Optn and Rab8 (211). Although the physiological function of Htt is unknown, it is found to interact with several factors involved in vesicle trafficking, including the Htt-associated protein 1 (213). In addition, *Optn* silencing or expression of p.Q398W and p.E478G pathological mutants in neuron-like NSC-34 cells is sufficient to block lysosome fusion to autophagosomes, resulting in autophagosome accumulation. (2) Neuroinflammation. Inflammation of the central nervous system, a process known as neuroinflammation, is one of the most important hallmarks of neurodegenerative diseases that occurs very early during the disease and is regulated by microglia. In the presence of endogenous stimuli, potentially triggered by aggregated proteins, activation of pro-inflammatory responses by the NF-κB pathway leads to neuron loss (214, 215). Interestingly, production of pro-inflammatory mediators by activated microglial cells is regulated by the mitochondrial dynamics: mitochondrial fission leads to ROS production and induction of pro-inflammatory cytokines through the activation of NF-κB and MAPK signaling pathways in LPS-stimulated microglial cells (216). It has been suggested that regulation of the NF-κB-dependent inflammatory pathway by Parkin might contribute to the neuroprotective properties of Parkin (217). Accordingly, *PARK2* knockout mice exhibited increased susceptibility to neuronal loss induced by LPS, an activator of the NF-κB pathway (218). The role played by Optn in NF-κB-dependent inflammation has been a matter of debate despite its structural homologies with NEMO. Indeed, although the C-terminal UBDs of NEMO and Optn are interchangeable, Optn cannot substitute for NEMO due to its inability to interact with the IKKα/β kinases required for NF-κB activation (50). In contrast, overexpression of Optn was shown to inhibit NF-κB activation as Optn could compete with NEMO for its interaction with ubiquitinated substrates through its UBD (206, 219). Data using CRISPR/Cas9-mediated *Optn*-KO cells are also consistent with an inhibitory role of Optn in NF-κB activation, although *in vivo* studies in mice have shown that Optn is not essential for the activation of NF-κB (220–222). More interestingly, reconstitution of these cells with ALS-associated mutants (Q398X and E478G) fails to inhibit NF-κB activity (38). The potential role of ALS mutations of Optn in NF-κB-mediated neuroinflammation would, thus, be interesting to explore, especially as Optn is highly expressed in brain (35).

#### Involvement of TBK1 in ALS

As for *Optn*, mutations of the *TBK1* gene were first linked to primary open glaucoma and NTG (223, 224). Using exome sequencing and targeted mutation screens, gain-of-function mutations of *TBK1* were discovered in ALS patients (203, 225–227). These human genetics studies have uncovered a link between *TBK1* and ALS in Swedish, Chinese, and Taiwanese populations and identified that nonsense and frameshift mutations decrease the expression of TBK1 at both mRNA and protein levels, while missense mutations do not. As all members of the IKK family, TBK1 contains four domains: a N-terminal serine/threonine kinase domain, an ubiquitin-like domain, and two C-terminal coiled-coil domains, CCD1 and CCD2. Mutations of the kinase and CCD1 domains account for a greater proportion of diseases than mutations of other domains (227). As TBK1 substrates include not only Optn but also p62 and NDP52, pathological mutations in its catalytic activity alter the phosphorylation of all these mitophagy receptors, while mutations in its CCD2 domain prevent mainly its interaction with Optn and p62. For instance, deletion of CCD2 (p.690-713del) that is responsible for the development of ALS, impairs TBK1 association with Optn suggesting that a loss of TBK1 and Optn binding may be important for the development of the disease (228). Various inclusions were found in different brain regions of ALS patients harboring TBK1 mutations. The nature of these inclusions, in particular the presence of TDP-43, p62, or ubiquitin, suggests that deregulation of TBK1 autophagic functions could lead to aggregated protein accumulation, which may be one of the pathophysiological mechanisms of the disease (181). As for *SOD1, VCP, UBQLN2*, and *p62*, mutations of *TBK1* genes (loss-of-function) have also been implicated in FTD (229).

### Involvement of Mitophagy in Parkinson’s Disease

Although Optn has not been directly associated with Parkinson disease, its presence was detected in pathological structures (Lewy bodies) present in Parkinson disease. Since Optn mediates its mitophagy function downstream of PINK1 and Parkin, which have been formally reported to be involved in Parkinsonism, we detailed here the genes responsible of mitochondrial dysfunctions in Parkinson disease and Parkinsonism.

#### Parkinson and Associated Genes

Parkinson’s disease is specifically manifested by the progressive degeneration of dopamine neurons in the substantia nigra of the brain. The disappearance of these cells is accompanied by disturbances of the neuronal networks associated with them in different areas of the brain: in the striatum, thalamus, and sub-thalamic nucleus. Parkinson’s disease is generally not a hereditary disease and most hereditary cases are sporadic with only 10% of familial cases. In addition, about 5% of genetic forms are linked to mutations affecting specific genes. Outstandingly, identification of the function of mutated genes in Parkinson’s disease has allowed a better understanding of autophagy. The current nomenclature refers to PARK as the chromosomal regions associated with Parkinson’s disease and over six genes have been formally associated with familial Parkinson diseases including *PARK1-4* (SNCA; α-synuclein), *PARK8* (leucine-rich repeat kinase enzyme) capable of transferring a phosphate group from one molecule to another to regulate its activity, *PARK2* (Parkin), *PARK6* (PINK1), *PARK7* (DJ-1), *PARK9* (ATP13A2), and also glucocerebrosidase, *PARK17* (VPS35), *PARK18* (EIF4G1), and *PARK16* (230). However, mutations of these genes are not systematically associated with the development of the disease. Links between Parkinson’s and mitochondria have been recently emphasized, since the pathological form of α-synuclein, a mitochondria-localized protein connected to the endoplasmic reticulum membrane, carries the necessary and sufficient information to trigger the disease (230).

#### Mitochondrial Dysfunction in Parkinson’s Disease

As the links between mitophagy-related proteins and Parkinson’s have been extensively described before [recent reviews include (231, 232)], we focused our attention on Parkin, PINK1, and Optn and their implication in Parkinson’s disease. Among the genes mutated in Parkinsonism, three autosomal inherited genes have been identified by Genome-Wide Association Studies to impact mitochondrial function: *PARK2* (Parkin), *PARK6* (PINK1), and *PARK7* (DJ-1) (231, 233). Parkin is the second largest gene of the genome containing 12 exons that encode a 455-amino acid protein. About half of the reported mutations of the Parkin gene affect exons 2–4 encoding the ubiquitin ligase domain, the linker region, and the beginning of RING0 domain. A deletion of exon 3 is the most frequent mutation of the Parkin gene. The second most frequent nucleotide change is the c.924>T mutation in exon 7 (234). Mutations in *PARK6* (PINK1) are the second most representative cause of autosomal recessive early-onset Parkinsonism. In contrast to *PARK2*, the majority of *PARK6* mutations are missense or nonsense mutations and rarely exon deletions. Since Parkinsonism specifically affects dopamine neurons, it suggests that the three proteins are not essential for general mitochondrial functions, but for more specialized ones. Surprisingly, *PARK2/6/7* triple knockout (KO) in mice does not alter dopaminergic neurons, although it affects mitochondrial functions (235, 236). On the other hand, KO of *PARK2* in *Drosophila* displays dramatic effects due to mitochondrial dysfunctions, since *PARK2*-deficient male and female flies are sterile, suffer from severe locomotor defects, and cannot fly (237, 238). The same type of muscle degeneration was observed in *PARK6*-deficient flies, and since their phenotype could be rescued by overexpressed Parkin, PINK1 was positioned upstream of Parkin (239, 240). Interestingly, patient mutations in both *PARK2* and *PARK6* genes prevent recruitment of Parkin to mitochondria indicating that mitochondrial dysfunction may play a role in sporadic Parkinson disease (94, 96, 97, 241). Although mutations of the *Optn* gene have not yet been identified in Parkinson’s patients, a link between Optn-associated mitophagy dysfunction and development of this disease is strongly suggested by Wise and Cannon (63). Indeed, using a pre-clinical Parkinson’s disease rat model, these authors showed that Optn is enriched in dopamine neurons of the midbrain, and that its expression is increased by rotenone treatment *in vivo*. More interestingly, in rotenone-treated animals, Optn colocalizes with LC3 and α-synuclein positive puncta.

### Physiological Relevance of Mitophagy in Cancer

Links between autophagy receptors and cancer is complex and sometimes even paradoxical. Depending on the stage of the tumor and the type of cancer, autophagy can prevent tumor initiation but can also be a factor of resistance to anti-tumor chemotherapy. In fact, effectors of autophagy such as Optn play such a fundamental role in cellular homeostasis that their mutations are rarely responsible for cancer, although the cellular processes in which they are involved are associated with the occurrence of cancers. Generally, tumors undergo a drastic metabolic reprogramming, since most of them carry mutations altering key enzymes of the tricarboxylic acid cycle (TCA) or the activity of the OxPhos system, thereby limiting these metabolic activities (242). Therefore, cancer cells use glycolysis as the main source for ATP production (“Warburg effect”). However, the connection between mitochondria and cancer cells is not only linked to metabolism but also to homeostasis. Mitophagy plays an important role in cellular homeostasis to ensure mitochondria maintenance and detoxification, since accumulation of defective mitochondria generates oxidative stress. Then, oxidative stress is responsible for DNA damage and inflammation, leading to chronic tissue damage and tumor initiation (243).

#### Alteration of Mitophagy-Associated Genes in Tumor Development

Various mitophagy effectors have been shown to be defective in human cancers, including PINK1, Parkin, Optn, p62, TAX1BP1, NIX (BNIP3), and TBK1. PINK1 expression is lost, downregulated, or mutated in glioblastoma, ovarian cancer, and neuroblastoma (244, 245). Parkin is localized at human chromosome 6q25-q26 that is frequently deleted in ovarian, breast, bladder, and lung cancer types (246). Also, consistent with its tumor suppressor function, Parkin KO mice develop hepatic tumors (247). It has been reported that the level of expression of Optn is downregulated in urothelial carcinoma (248). Interestingly, ubiquitination of Optn by tumor-suppressor E3 Ub ligase HACE1 activates autophagy through promoting the physical interaction between the autophagy receptors Optn and p62 (64). They further demonstrate that the HACE1-Optn axis suppresses growth and tumorigenicity of lung cancer cells. Thus, the interaction of Optn and p62 seems to suppress tumorigenicity. Interestingly, recent *in vivo* data highlight the link between HACE1 and neurodegenerative diseases, since loss of HACE1 impacts Huntington disease-like phenotypes in a mouse model, and reduced levels of HACE1 were observed in patients (249). Other studies have shown that the autophagy receptor p62 is a critical target of Ras-induced tumorigenesis. Indeed, it was shown that the loss of p62 in embryo fibroblasts enhances Ras-activated cell death, reducing oncogenic transformation (250). The inability of autophagy-defective tumors to eliminate p62 contributes to oxidative stress and DNA damage resulting in tumor initiation. Many studies have shown that p62 is dramatically upregulated in Beclin^+/−^ tissues and in human tumors (251, 252). Analysis of the *TAX1BP1* gene reveals a polymorphism in head and neck cancer patients (253). BNIP3 and BNIP3L are both upregulated in pre-malignant ductal carcinoma *in situ* breast cancer. However, BNIP3 expression is lost in invasive breast tumors, which correlates with lymph nodes metastasis and poor prognosis (254). BNIP3 is also lost in 59% of pancreatic cancer and corresponds to poor survival, and its gene is silenced through aberrant methylation in chemoresistance of colorectal cancer to 5-fluorouracil and pancreatic cells (255–258). Mouse models also support a tumor suppressive role for *BNIP3*, since BNIP3 KO promotes tumor growth (259). Several studies suggest a role of TBK1 in cancer. First, it was shown that TBK1 is highly expressed in lung, breast, and colon cancer and is mutated (P675L) in lung carcinoma (260, 261). Furthermore, it was reported that TBK1 is required in KRAS-dependent cell transformation. A major breakthrough was the discovery that TBK1 is chronically activated in a variety of cancer cells, and that its activity is required for RAS-induced transformation (262). Recently, it was shown that TBK1 is upregulated in bladder cancers, and that its pharmacological inhibition dampens cell proliferation and migration (263). Since Optn is downregulated in urothelial carcinoma, the most common type of bladder cancer, and may act as an inhibitor of TBK1 activity (264), it will be interesting to determine whether the lack of expression of Optn is responsible for increased TBK1 activity, and thus could be a relevant target for the treatment of these cancers. It was further shown that mitophagy is required for cell transformation during the later stages of oncogenesis. Indeed, mitophagy has been involved in KRAS-induced transformation to overcome an energy deficit in tumor cells and is also required for benign hepatic tumors to progress into malignant hepatocellular carcinoma (265). In this last case, p53 is degraded during mitophagy to prevent its nuclear function, through its phosphorylation by PINK1 and, as a consequence, *p53* gene invalidation mimics PINK1-related pro-autophagic responses and increases the expression of Optn, p62, and NDP52 (266, 267). Finally, alteration of tumor fate from adenomas/carcinomas to benign oncocytomas has been linked to upregulated KRAS-induced autophagy and concomitant accumulation of dysfunctional mitochondria due to loss of ATG7 (268).

#### Biological Functions of Mitophagy-Associated Genes in Cancer

Different cellular processes related to mitophagic effectors may also explain the link between mitophagy and cellular transformation. Indeed, mitochondrial dysfunction and mitophagic effectors can act on tumor development at several levels: cell cycle, production of ROS, inflammation, UPRmt, and apoptosis.

Interestingly, Optn, p62, TBK1, PINK1, and Parkin are all involved in cell cycle regulatory mechanisms, in addition to their role in mitophagy. Indeed, Optn specifically controls the activity of a mitotic enzyme, Polo-like kinase 1 (PLK1) that has been linked to the development of several types of cancer when its activity is deregulated (269). Other studies demonstrated that p62, expression of which is regulated by PLK1, is phosphorylated by Cdk1 to maintain appropriate Cyclin B1 levels and to allow proper entry and exit from mitosis (270, 271). In search of TBK1 substrates responsible for survival in lung carcinoma, it was found that TBK1 phosphorylates the mitotic kinase PLK1, suggesting that TBK1 could regulate mitosis to drive lung cancer cell survival (272). Since TBK1 and PLK1 target the same site of phosphorylation on Optn (Ser177) that is required to inactivate PLK1 (269), it will be important to determine whether Optn could be involved in TBK1 regulation of mitosis in connection with its cancer cell survival activity. In addition to TBK1, PINK1 and Parkin have been shown to regulate mitotic progression (273), suggesting a relationship between mitochondrial integrity and the cell cycle. It is, therefore, difficult to determine whether the involvement of these proteins in cancer is due to their role in the cell cycle, in mitophagy, or in both cellular processes. Production of ROS can regulate mitophagy through AMPK, which collapses mitochondrial membrane potential and increases the number of dysfunctional mitochondria (274). In fact, AMPK promotes mitophagy through activating ULK1 and inhibiting mTORC1. On the other hand, damaged mitochondria are the major source of ROS, and since their accumulation are tumorigenic; mitophagy is considered as a tumor suppressor mechanism (275). Cancer cells induce oxidative stress leading to high mitophagy activity in the tumor microenvironment. This increases production of nutrients that bring energy to the transformed cells and ROS that increase genomic instability of cancer cells.

As for neurodegenerative diseases, prolonged acute inflammation that may be generated, for example, by DAMP-induced mitochondrial stress, can also initiate cancer (276). Mitophagy in macrophages was shown to counteract this dangerous process. Three mitophagy-related genes have been associated with the development of inflammatory bowel diseases such as Crohn’s disease and ulcerative colitis that are associated with an increased risk of developing colorectal cancer. First, the *Autophagy related 16-like 1* (ATG16L1) gene that maps to a Crohn’s disease susceptibility locus encodes a protein associated with depolarized mitochondria in response to mitophagy inducers, although its association with Crohn’s disease seems rather due to its function in xenophagy (62, 277). Second, Optn expression deficiency was observed in 10% of Crohn’s disease patients and Optn KO in mouse BMDMs results in reduced proinflammatory cytokine secretion and confers susceptibility to *Citrobacter*-induced colitis (278, 279). Finally, a missense mutation of NDP52 in the middle coiled-coil region is linked to Crohn’s disease type of inflammatory bowel disease (280). In addition, pancreatitis (severe pancreas atrophy, fibrosis, and inflammation) that can result from disruption of genes involved in autophagosome formation or lysosomal function increases the risk of cancer (281). Interestingly, pancreatitis induces mitochondrial depolarization and fragmentation and, therefore, mitophagy, reducing the deleterious effects of inflammation.

The mitochondrial unfolded protein response (UPR^mt^) is a protective transcriptional response that promotes survival of mitochondria in case of accumulation of misfolded mitochondrial proteins. Recently, it was found that not only mitochondrial misfolded proteins are degraded by the UPR^mt^ but also that cytosolic proteins that are prone to aggregation are imported into the mitochondria for degradation (282). UPR^mt^ is disrupted in multiple pathologic states, and it is suggested that regulatory changes in UPR^mt^ may be involved in tumor development (283). It was first shown that introduction of mtDNA from a metastatic into a non-metastatic cell line was sufficient to confer metastatic capacity to the non-metastatic cells (284). Indeed, mutations in mtDNA can lead to a defect in OxPhos and accumulation of ROS, which can in turn lead to the oxidation of proteins and their misfolding. Cancer cells, therefore, are predicted to rely on the UPR^mt^ to survive such stress (285). Since polarized mitochondria harboring misfolded proteins accumulate PINK-activated Parkin in foci with ubiquitin, Optn, and LC3 (286), it is tempting to speculate that one or several of these proteins may act, in this case, as pro-oncogenic agents by facilitating tumor cell survival through activation of the UPR^mt^.

## Conclusion on the Involvement of Optn-Mediated Mitophagy in Pathologies

In addition to neurodegenerative disorders and cancers, mitophagy is also involved in aging, heart, and liver diseases, although the mitophagy-related role of Optn in these diseases, as well as in aging and obesity, has not yet been explored. However, a recent study showed that Optn-mediated autophagy plays a crucial role in high glucose-induced renal tubular epithelial cells senescence in diabetic nephropathies (287). In this pathology, 50% of renal tubular cells exhibit fragmented mitochondria, and insufficient mitophagy in these cells leads to cellular senescence. By acting on mitophagy, Optn may, therefore, be a potential anti-senescence factor in diabetic nephropathies. Thus, it is not surprising that Optn expression correlates with the progression of diabetic nephropathy. This finding implies that patients with autophagy-associated neurodegenerative disease may be more prone to develop diabetic nephropathy. While it is not the case for ALS, diabetes has been associated with higher risk of Parkinson’s disease (288, 289). With the increasing amounts of data accumulated over the past 5 years on the detailed mechanisms of Parkin-dependent mitophagy, research has focused on the involvement of their components, and especially the mitophagy receptors and their regulators, in neurodegenerative diseases. Although links between Optn, mitophagy, and ALS have been identified, the role of Optn in the development of Parkinson’s disease has not yet been established and no mutations of *Optn* responsible for this disease have yet been identified, despite the presence of Optn in inclusion bodies of both diseases. In contrast, relationships between *Optn* mutations and different forms of glaucoma (OAG and TNG) are well established, but the role of autophagy/mitophagy in the development of these pathologies is unclear and depends on the experimental models used. Better understanding of the involvement of mitophagy in these degenerative diseases requires generation of animal models. An interesting mouse model that remains to be established will be to cross existing mt-Keima mice that were established to measure mitophagy efficiency *in vivo* (290) with mice models reproducing neurodegenerative diseases such as ALS (i.e., A53T α-synuclein transgenic mice). Identification of Optn as an autophagy receptor has undoubtedly uncovered critical functions of Optn in cell homeostasis and opens new exciting avenues for further research in the field of neurodegenerative diseases. Linking the biological function(s) of Optn to its associated pathologies is the next challenge. Interestingly, pathology-associated mutations of Optn, mainly linked to neurologic tissues, do not appear to cause any disorder in other tissues, despite the wide distribution of Optn expression. How this cell type specificity is achieved is also still to be understood. Transgenic and cell-type-specific knockout animal models are, therefore, needed to explore the role of Optn in normal cellular functions, to identify the mechanisms of pathogenesis of Optn-associated diseases, and to understand how deregulation of the Optn-dependent mitophagy could initiate or promote development of these diseases.

## Author Contributions

RW, EL, and PG are the major contributors of the review: conception, writing, and illustrations. SC participates to the writing and figure/table composition of the review.

## Conflict of Interest Statement

The authors declare that the research was conducted in the absence of any commercial or financial relationships that could be construed as a potential conflict of interest.
